# Comparing Models of the Children’s Yale-Brown Obsessive-Compulsive Scale (CY-BOCS) in an Italian Clinical Sample

**DOI:** 10.3389/fpsyt.2020.00615

**Published:** 2020-08-06

**Authors:** Caterina Novara, Susanna Pardini, Francesco Cardona, Massimiliano Pastore

**Affiliations:** ^1^Dipartimento di Psicologia Generale, Università di Padova, Padova, Italy; ^2^Dipartimento di Neuroscienze Umane, Università di Roma “La Sapienza”, Roma, Italy; ^3^Dipartimento di Psicologia dello Sviluppo e della Socializzazione, Università di Padova, Padova, Italy

**Keywords:** Children’s Yale-Brown Obsessive-Compulsive Scale (CY-BOCS), Obsessive-Compulsive Disorder, Tourette Syndrome, TIC Disorder, Bayesian model comparison

## Abstract

**Background:**

Obsessive-Compulsive Disorder (OCD) is a mental disorder that interferes with daily functioning and may arise during childhood. The current study is the first attempt by Italian researchers to validate the Children’s Yale-Brown Obsessive-Compulsive Scale (CY-BOCS).

**Aims:**

The study’s primary aim was to investigate the best CY-BOCS model fit, adopting a Bayesian model comparison strategy, among four different factor models: a one-factor model; a two-factor model based on Obsessions and Compulsions; Storch et al.’s and Mc Kay et al.’s two-factor model based on Disturbance and Severity. The study also aimed to investigate the types of treatments found in a sample of Italian OCD children patients.

**Methods:**

The study sample was made up of 53 children with OCD and 14 children with Tourette Syndrome and TIC.

**Results:**

An analysis of our data demonstrated that the Obsessions and Compulsions model was the most plausible one, as it demonstrated the best fit indices, strong convergent validity, and good reliability. The study results additionally uncovered that 24.5% of the children in the OCD sample had not yet begun any treatment pathway a year after a diagnosis was formulated.

**Conclusions:**

These findings suggest that the Obsessions and Compulsions scales of the CY-BOCS separately represent appropriate instruments to evaluate children with OCD.

## Background

### Prevalence and Phenomenology

The estimated lifetime prevalence of Obsessive-Compulsive Disorder (OCD) ranges between 2 and 4% (1–3). On average, symptoms of OCD present at 19.5 years of age (between 10 and 40); in males, the onset is generally before the age of 10, while in females it is during or after adolescence (usually between 20 and 25) (4, 5).

The estimated prevalence of OCD in childhood and adolescence is 0.25%–4%, with those aged between 16 and 18 years old (1%) showing the highest prevalence. The disorder is frequently characterized by gradual onset, a chronic course, and exacerbation of symptoms over a long period of time (the average length of time is 8.9 years over a lifetime) (5–7). Most juvenile OCD patients show a progressive age-related worsening of symptoms, and poor school performance seems to be associated with symptom severity (8). According to several studies, juvenile OCD seems to be mainly characterized by compulsions alone (4, 9, 10) and may be difficult to differentiate from tic-like behaviors.

Garcia etal. (11) more recently reported that 96% of an OCD juvenile patient sample had a mean of four concomitant compulsions, 75% had two concomitant obsessions, and 18% reported having compulsions without obsessions.

Moreover, several researchers put in evidence that juvenile OCD is left unrecognized or untreated and, for this reason, it is characterized by an insidious and progressive course; it can severely disrupt global functioning, negatively affect the lives of patients and their families, and persist in the course of later childhood, adolescence, and adulthood (i.e., 12–15).

### Comorbidity

OCD in childhood is frequently associated with other psychiatric conditions such as depression and anxiety disorders, attention deficit hyperactivity disorder (ADHD), tic disorder (TIC), and autism spectrum disorder (in 77-85% of cases), and that can further exacerbate the patient’s condition. Some studies have uncovered overlapping comorbidities such as TIC and Tourette Syndrome (TS), which are found in 9-59% of individuals with OCD (16). The presence of OCD has, in turn, frequently been observed in children who have been diagnosed with TIC. One study, in fact, reported that more than 50% of children with TIC also manifested OCD symptoms (17). Bloch etal. (18) pointed out that in approximately 30−50% of the sample of children with TS, it was in comorbidity with OCD. The best estimate of the prevalence of TD in school-age children is approximately 3–8/1000 (19). Boys seem to be more likely than girls to manifest TIC, with a gender ratio ranging between 2:1 and 4:1. The prevalence of TS seems to decrease when children/adolescents grow older, with the highest prevalence found in 7–10-year-olds (20). OCD symptoms associated with TIC generally present at a pre-pubertal age and may predate the onset of TIC (21).

### The Psychometric Properties of the CY-BOCS

Several studies have highlighted the importance of investigating childhood-onset OCD, as it represents a serious mental health problem often associated with other conditions that may have important implications for patients’ current and future health and quality of life. The Children’s Yale-Brown Obsessive-Compulsive Scale (CY-BOCS; 22, 23) is currently the instrument of choice to assess the presence and severity of OCD symptoms in children/adolescents and to monitor treatment. To our knowledge, no attempt has been made to validate the Italian version of the scale. The CY-BOCS is a semi-structured interview made up of 10 items rated on a 5-point Likert scale evaluating the severity of Obsessions and Compulsions across five dimensions, Frequency, Interference, Distress, Resistance, and Control, during the previous week and up to the time of interview. A score above 16 is generally considered indicative of the presence of OCD (16-23 = moderate severity; 24-40 = severe).

The CY-BOCS is similar to the adult version (Y-BOCS), and numerous studies suggest that both can be explained by a two-factor model: Obsessions and Compulsions. In contrast, the validity of a three-factor model (the Obsessions and Compulsions scores and the total severity score) has not been confirmed (24–27).

Other studies have found evidence confirming the validity of a two-factor model based on a Disturbance factor and a Severity factor in an adult (24, 27, 28) and juvenile population (29, 30). Using Confirmatory Factor Analysis (CFA), McKay etal. (29) examined a Severity factor related to general impairment and reflected in Distress, Interference, and Frequency symptoms; the Disturbance factor was linked to the Resistance and Controlling symptoms of both Obsessions and Compulsions. These results were partially replicated by Storch etal. (31), who examined the same two-factor model, the only difference being that the symptom frequency items were loaded onto the Severity factor rather than on the Disturbance one.

In light of conflicting results and in view of the importance of an appropriate assessment of the disorder in juvenile populations, the current study’s primary aim was to use a Bayesian model comparison strategy to compare three two-factor models and one single-factor model to determine the best model fit for the CY-BOCS in a sample of Italian children/adolescents diagnosed with OCD.

The single-factor model consisted of only one factor that included all the symptoms related to Obsessions and Compulsions. The second consisted of a two-factor model examining Obsessions and Compulsions. The other two two-factor models were both based on Severity and Disturbance; they differed only with regard to the frequency variable, as in one model, it was loaded onto the Severity factor, while in the other, it was loaded onto the Disturbance one.

Given the shortage of studies examining this disorder in Italian children/adolescents, the study also focused on collecting information regarding the severity of disability and the kind of treatment that is provided in different Italian public health facilities.

## Methods

### Participants

The study sample was made up of 67 children/adolescent outpatients/inpatients recruited at different public health facilities located in Northern (n=40), Southern (n=12), and Central (n=15) Italian towns/cities between 2014 and 2017. All of the children/adolescents were evaluated by Pediatric Neuro-Psychiatrist specialists who formulated their diagnoses on the basis of the patient’s score on the Diagnostic and Statistical Manual of Mental Disorders, Fifth Edition (DSM-5; 19). Out of the 67-patient population enrolled in the study, 53 were diagnosed with OCD (79.1%), 11 were diagnosed with TS (16.4%), and 3 were diagnosed with TIC (4.5%).

### The OCD Group

The OCD study group was made up of 33 boys (62.3%) and 20 girls (37.7%) (mean age = 12.9 years; SD = 3.3 years, range = 6 to 18 years). On average, they were 11.4 years old (SD = 3.1 years, range = 4 to 16 years) when OCD was diagnosed, and they were first evaluated approximately 1.5 years after the first symptoms of the disorder presented (SD = 1.5 years, range = 0 to 6 years).

Comorbid conditions, principally TIC, ADHD/Attentive Disorder, Learning Disabilities, and Anxiety/Emotional Disorder, were present in 52.8% of the sample. Moreover, 15.1% had at least one family member who had been diagnosed with OCD, TIC, or TS. Out of the total patient group being examined, 60.3% were undergoing treatment (26.4% pharmacological treatment, 7.5% psychological treatment, 26.4% both). Out of the total group, 50.9% had never been hospitalized for the disorder, and 24.5% were receiving some type of psychological treatment. As far as school was concerned, 9.5% were receiving some type of educational support, and 3.8% had already left school. Overall, the average number of years of schooling in this group was 7.2 years (DS: 3.5; range: 0 to 13 years).

### The Tourette Syndrome and TIC Disorder Group

The second study group was made up of 8 boys (73%) and 3 girls (27%) who were diagnosed with TS and 2 boys (67%) and 1 girl (33%) who were diagnosed with TIC. The mean ages in the TS and TIC subgroups were, respectively, 10.4 years (SD = 2.3, range = 8 to 16 years) and 9 years (SD = 2, range = 7 to 11 years). On average, they were 6.6 years old (SD= 1.5, range=4 to 9 years) when they were diagnosed. The TS patients were evaluated approximately 3.7 years after the first symptoms presented (SD = 2.9 years; range = 1 to 10 years), and the TIC patients were first evaluated less than a year after the first symptoms presented. Comorbid conditions, for the most part, OCD/obsessive-compulsive traits, ADHD, separation anxiety disorder, and generalized anxiety disorder/depressive traits, were present in 82% of the TS patients and in all of the TIC patients.

As far as the TS subgroup was concerned, 55% had a family history of OCD (18%), TIC, or TS (36%). Twenty-seven percent were undergoing treatment (9% pharmacological treatment, 9% psychological treatment, and 9% both). None of the patients in this group had ever been hospitalized for the disorder. Two of the TIC patients had a family history respectively of OCD and TS; none were undergoing treatment, and none had ever been hospitalized for the disorder. As far as school was concerned, one patient was receiving special education assistance. Overall, the average number of years of schooling was 4.2 years (SD: 2.3 years; range: 2 to 10 years) in the TS subgroup and 3 years (SD: 2 years; range: 1 to 5 years) in the TIC subgroup. The participants’ demographic data are outlined in **Table 1**.

**Table 1:** Demographic characteristics of the patients studied.

	OCD groupN=53	Tourette Syndrome & TIC groupN=14
**Gender** (% of male)	33 (62.3%)	10 (71.4%)
**Age** M (SD)	12.9 (3.3) min. = 6; max. = 18	10.1 (2.3) min. = 7; max. = 16
**Years of schooling** M (SD)	7.2 (3.5) min. = 0; max. = 13	3.9 (2.2) min. = 1; max. = 10
**Students** Student Student with scholastic support Special course School drop-out	46 (86.8%) 3 (5.7%) 2 (3.8%) 2 (3.8%)	13 (92.9%) 1 (7.1%) 0% 0%
**Age at diagnosis** M (SD)	11.4 (3.1) min. = 4; max. = 16	7.1 (1.8) min. = 4; max. = 11
**Comorbidity** (%) TIC/Tourette Anxiety symptoms Mood disorder Learning disorder ADHD OCD traits	28 (52.8%) 6 (11.3%) 8 (15.1%) 6 (11.3%) 6 (11.3%) 2 (3.8%) /	12 (85.7%) / 3 (21.4%) 0 0 4 (28.6%) 5 (35.7%)
**Familiarity** (%)	8 (15.1%)	8 (57.1%)
**Type of treatment** Pharmacological treatment (%) Psychological treatment (%) Pharmacological and psychological treatment (%) No treatment after one year from the diagnosis (%)	14 (26.4%) 4 (7.5%) 14 (26.4%) 13 (24.5%)	1 (7.1%) 1 (7.1%) 1 (7.1%) –
**Hospitalized** (%)	4 (7.5%)	0%
**Hospitalized in the past** (%)	9 (17%)	0%

N, number of group individuals; M, Mean; SD, Standard Deviation; min., Minimum; max., Maximum; OCD, Obsessive-Compulsive Disorder; ADHD, Attention Deficit Hyperactivity Disorder.

## Procedures

During the first phase of the study, three specialized psychologists were trained in administering the CY-BOCS. The training sessions were audio-recorded and evaluated by the first author of the present study. The assessment phase was carried out at the different mental health facilities respectively located in the north, center, and south of Italy. All the outpatients and inpatients were diagnosed by specialized Pediatric Neuropsychiatrists in those facilities using the Diagnostic and Statistical Manual of Mental Disorders, Fifth Edition (DSM-5).

The inclusion criterion consisted of a primary diagnosis of OCD and/or TS and/or TIC in a child/adolescent, and all consecutive patients assessed for the first time or during the ongoing monitoring visit were included.

Individuals with neurological disorders or mental retardation based on a previous Pediatric Neuro-Psychiatrist assessment were excluded. Once the patients were identified, they were interviewed individually using the CY-BOCS (or in their parents’ presence if they were 7 years old or younger). The interview was carried out by one of the specialized psychologists who was unaware of the patient’s diagnoses. The psychologists were simply informed that the patients they would be interviewing could have OCD or/and TIC or/and TS. The interview lasted approximately 40 min.

The Obsessive-Compulsive Inventory-Child Version (OCI-CV) was also administered, with parents if the child was less than 7 years of age.

This study was conducted in accordance with the Declaration of Helsinki and approved by the Ethical Committees of the Department of General Psychology (University of Padova) and all of the mental health facilities participating in the study. All of the children/adolescents participated on an entirely voluntary basis and were enrolled only after their parents had signed consent forms. Their social and demographic information were collected after they were enrolled.

### Measures

The *Children’s Yale-Brown Obsessive-Compulsive Scale* (22, 23) was the main measure under consideration. Although the original version of the CY-BOCS was translated with the forward translation mode into Italian (32), to our knowledge, to date, no study has investigated its psychometric properties in Italian juvenile patients.

The *Obsessive-Compulsive Inventory-Child Version* (OCI-CV; 33, 34) is a well-established 21-item self-report questionnaire using a three-point Likert scale (ranging from 0 to 2) to assess the frequency of obsessions and compulsions over the previous month. Its six sub-scales concern Doubting/Checking, Obsessing, Hoarding, Washing, Ordering, and Neutralizing. The questionnaire has demonstrated good/modest internal consistency (33, 35). One study by researchers assessing Italian patients reported an excellent/good/acceptable internal consistency both for the inventory’s total score and sub-scale scores (34). In our sample of patients, the internal consistency of the total score was found to be good (Cronbach’s alpha= 0.84).

The *Child Behavior Checklist 6-18* (CBCL/6-18; 36; Italian version by A. Frigerio - IRCCS EUGENIO MEDEA-LA NOSTRA FAMIGLIA) is a standardized self-report questionnaire used by parents and teachers to screen for psychological problems (emotional and behavioral) and social competencies in children and adolescents. The questionnaire is formed of two sections regarding social competence/adaptive functioning and emotional/behavioral problems. The first section contains 20 items examining social and school activities and specifically the time devoted to sports, games/hobbies, types of activities engaged in, number of friends in general and of close friends, frequency of social interactions, level of self-sufficiency during playtime, problems at school, and academic performance. The second section, termed the “syndrome scale,” includes 113 items and uses a three-point Likert scale. The items are grouped into eight categories: anxious/depressed, withdrawn/depressed, somatic complaints, social problems, thought problems, attention problems, rule-breaking behavior, and aggressive behavior. Many studies have demonstrated a high rate of reliability between the CBCL scales and the psychological diagnoses formulated (37). The instrument is known to have a good test-retest reliability (ranging from 0.82 to 0.90) and internal consistency (Cronbach’s alphas ranged from 0.72 to 0.97). It also has strong criterion-related validity (36).

## Statistical Analysis

The Bayesian approach, which presents many practical advantages (e.g., 38–44), was used to analyze our data. A Bayesian model, in fact, provides an adaptive tool that is useful for handling small sample size by including prior information (45). Moreover, it provides a direct representation of the most credible values of the estimated parameter (44, 46) All analyses were performed using R statistical software (47). Each model was fitted using the Bayesian Markov Chain Monte Carlo (MCMC) estimation method implemented in the Just another Gibbs sampler (JAGS) package (48) coupled with the R statistical packages blavaan (49) and runjags (50).

Posterior distributions for each parameter were estimated using four MCMC chains, each running at least for 5000 replicates. MCMC convergence was assessed by calculating the potential scale reduction factor (PSRF) (also called Rhat; 51), which compares the ratio of the average variance of samples within each chain with the variance of the pooled samples across the chains; if all of the chains are at equilibrium, these will be the same, and R̂ will be one.

We adopted a model comparison strategy (52) in order to identify the best model by considering the following fit indices: the Bayesian Comparative Fit Index (BCFI), the Bayesian Tucker-Lewis Fit Index (BTLFI), the Bayesian Root Mean Square Error of Approximation (BRMSEA) (53), the Standardized Root Mean Square Residual (SRMR; 54), the Bayes Factor (BF), the Widely-Applicable Information Criterion (WAIC; 55), and the Akaike Weights (AW) (56–58). For each fit index and estimated parameter, we computed the mean of the posterior distributions as the estimate and the 90% credibility interval (also called Highest Posterior Density Interval; HPDI; 58–60).

**Figure 1:**
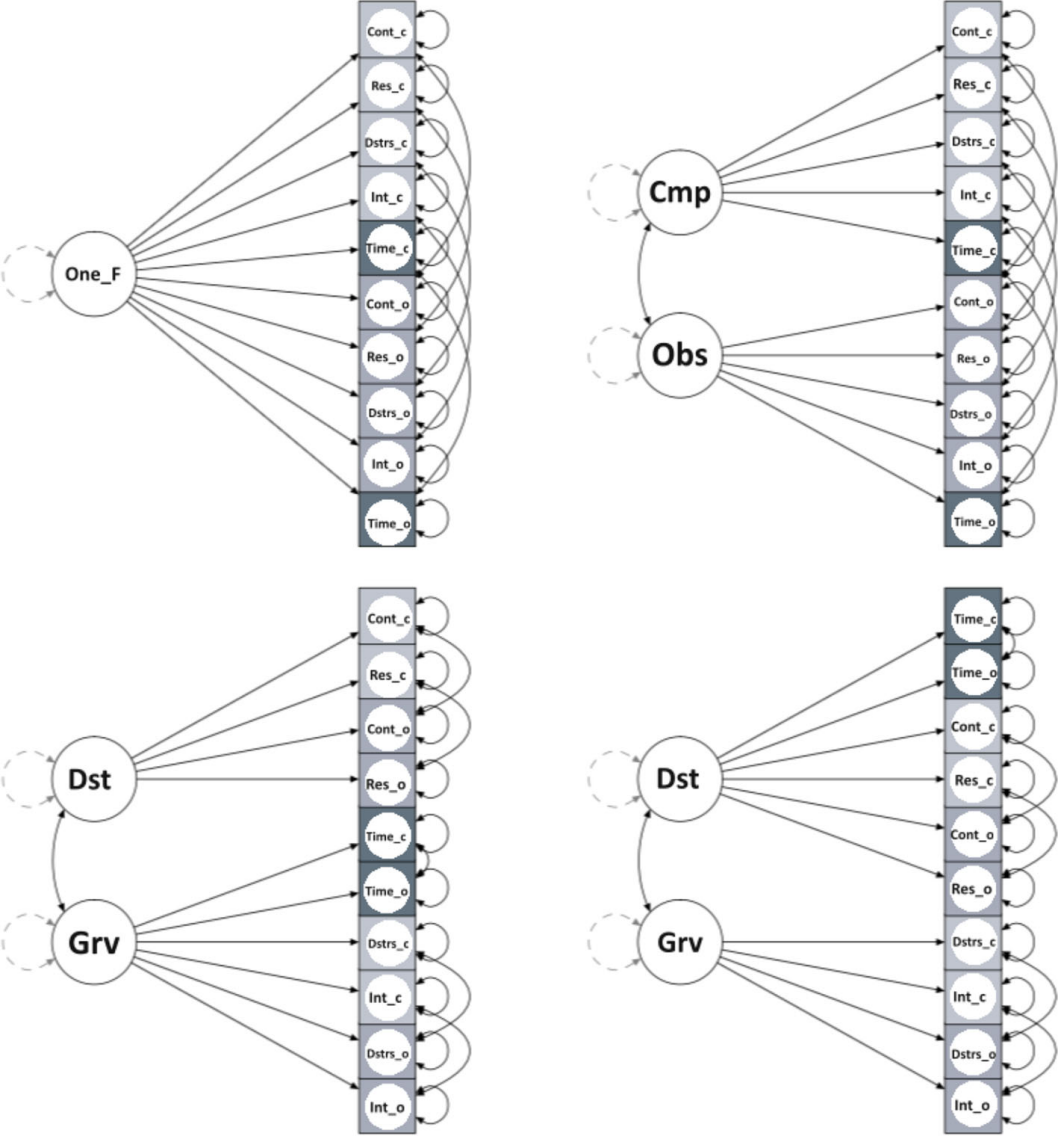
One_F, One-Factor; Cmp, Compulsions; Obs, Obsessions; Dst, Disturbance; Grv, Gravity; Cont_c, Control_compulsions; Cont_o, Control_obsessions; Res_c, Resistance_compulsions; Res_o, Resistance_obsessions; Dstrs_c, Distress_compulsions; Dstrs_o, Distress_obsessions; Int_c, Interference_compulsions; Int_o, Interference_obsessions; Time_c, Time occupied_compulsions; Time_o, Time occupied_obsessions.

We adopted the following priors for model parameters: 1) ν ∼ Normal(0, 31.6), for intercepts; 2) λ ∼ Normal(0.5, 0.58), for factor loadings; 3) θ ∼ Gamma(1, 0.5), for residual variances; 4) ϕ ∼ Beta(1, 1), for factor correlations. As suggested by Muthén and Asparouhov (45), we included informative small-variance priors for the cross-loadings, λc ∼ Normal(0, 0.32). In addition, we considered paired items by including residual covariance with prior distribution Beta(1, 1). The following models were considered: 1) a one-factor model; 2) a two-factor model (Obsessions and Compulsions); 3) Storch etal.’s (30) two-factor model; 4) McKay et al.’s (29) two-factor model (**Figure 1**). We also used Cronbach alpha and Pearson’s r for correlational analysis to assess reliability and construct validity, respectively.

## Results

### Comparing the CY-BOCS Scale With the Four Factor Models

The Bayesian Comparative Fit Index (BCFI; with 90% hpdi), the Bayesian Tucker-Lewis Index (BTLI, with 90% hpdi), the Bayesian Root Mean Square Error of Approximation (BRMSEA, with 90% hpdi), the Standardized Root Mean Square Residual (SRMR, with 90% hpdi), the Bayes Factor (log) (evidence regarding the worst model, in this case, the one-factor one), the Widely Applicable Information Criterion (WAIC), and the Akaike weight (56–58) were used as fit indices to examine the models. The BCFI, BTLI, and BRMSEA can range between 0 and 1, and values close to 1 on the BCFI and BTLI and close to 0 on the BRMSEA and SRMR indicate a good fit. The BF, which is the likelihood ratio of the marginal likelihood for two competing models, can be interpreted as the relative evidence or plausibility of one model with respect to another. The WAIC is a generalized version of the Akaike Information Criterion (AIC); the smaller the value, the better the model fits the data. Finally, the Akaike Weights represent an estimate of the probability that the model will make the best prediction on new data conditional on the set of models considered (56–58).

The fit indices of the models considered are outlined in **Table 2**. The single-factor model was found to have the worst fit, as it had a BF and an Akaike Weight equal to 0. The model formed by the Obsessions and Compulsions scales was found to be the most plausible, as it had a BF equal to 22.42 and an Akaike weight equal to 0.92. Besides presenting inferior fits in all the indices considered with respect to the Obsessions and Compulsions model, the other two competing models had nearly equivalent BF values (9.86 and 9.99 respectively) and predictions of new data (Akaike Weights 0.05 and 0.03). The Obsessions and Compulsions model can be considered 20.42 (0.92/0.05) times more plausible than Storch et al.’s model and 29.57 (0.92/0.03) times more likely to be correct than McKay’s model.

**Table 2:** Comparison of the fit indices of the models considered.

	npar	CC	BCFI	BCFI.hpd	BTLI	BTLI.hpd	BRMSEA	BRMSEA.hpd	SRMR	SRMR.hpd	BF	waic	se_waic	weight
**Model 1** **one factor**	35	100	0,73	(0.54; 0.9)	0,55	(0.24; 0.83)	0,16	(0.11; 0.21)	0,26	(0.25; 0.27)	0	1485,60	(43.9)	0,00
**Model 2** **Obs & cmp**	46	100	**0,98**	(0.92; 1)	**1,13**	(0.76; 1.52)	**0,02**	(0; 0.1)	**0,13**	(0.1; 0.16)	**22,42**	**1437,60**	(38.5)	0,92
**Model 3** **Storch et al**	46	100	0,97	(0.88; 1)	1,04	(0.65; 1.43)	0,04	(0; 0.12)	0,14	(0.11; 0.17)	9,86	1443,63	(40.6)	0,05
**Model 4** **McKay et al**	46	100	0,96	(0.87; 1)	1,04	(0.62; 1.45)	0,04	(0; 0.13)	0,14	(0.11; 0.17)	9,99	1444,37	(40.7)	0,03

Fit indices of the models considered (20000 mcmc posterior samples); NPAR, number of parameters; CC, Convergence criterion (a value less than 100 means that the model does not converge); BCFI, Bayesian Comparative Fit Index (with 90% hpdi); BTLI, Bayesian Tucker-Lewis Index (with 90% hpdi); BRMSEA, Bayesian Root Mean Square Error of Approximation (with 90% hpdi); SRMR, Standardized Root Mean Square Residual (with 90% hpdi); BF, (log) Bayes Factor (evidence with respect to the worst model, in this case, the one-factor model); WAIC, Widely Applicable Information Criterion; WEIGHT, Akaike weight; Obs&Cmp, Obsessions and Compulsions (52, 56, 58).

Bold text highlights the best fit indices.

**Table 3** outlines the estimated factor loadings for the Obsessions and Compulsions model. Every item is grouped in the corresponding scale. Loadings related to the Obsessions were all greater than or equal to 0.70; the loadings related to the Compulsions were all greater than or equal to 0.60. The cross-loadings were proximal to zero, and since the Highest Posterior Density Intervals (HPDs) of all the cross-loadings included zero, we can consider them irrelevant. We can assume, therefore, that they were more likely 95% of the time.

**Table 3:** The estimated factor loadings for the Obsessions and Compulsions model.

	OBSESSIONS	COMPULSIONS
**OBSESSIONS_TIME OCCUPIED**	**0,70** (0.45; 0.92)	-0,03 (-0.3; 0.26)
**OBSESSIONS_INTERFERENCE**	**0,80** (0.5; 1.1)	0,26 (-0.05; 0.57)
**OBSESSIONS_DISTRESS**	**0,85** (0.6; 1.13)	0 (-0.31; 0.3)
**OBSESSIONS_RESISTANCE**	**0,70** (0.4; 0.99)	-0,22 (-0.5; 0.08)
**OBSESSIONS_CONTROL**	**0,83** (0.54; 1.11)	-0,08 (-0.39; 0.23)
**COMPULSIONS_TIME OCCUPIED**	0,18 (-0.08; 0.44)	**0,60** (0.38; 0.83)
**COMPULSIONS_INTERFERENCE**	0,17 (-0.11; 0.47)	**0,65** (0.37; 0.9)
**COMPULSIONS_DISTRESS**	0,06 (-0.26; 0.37)	**0,75** (0.5; 0.99)
**COMPULSIONS_RESISTANCE**	-0,14 (-0.47; 0.21)	**0,87** (0.57; 1.15)
**COMPULSIONS_CONTROL**	-0,19 (-0.5; 0.1)	**0,75** (0.52; 0.99)

The best indices are marked in bold. The Highest posterior density interval (HPD) cross-loading parameters for the model are indicated in brackets.

### Discriminant Power, the Intercorrelation Between Scales, and Construct Validity

The scores on the CY-BOCS and the patients’ diagnoses were compared. The scale’s Sensitivity (Se; the proportion of true positives) and Specificity (Sp; the proportion of true negatives) was established by comparing the diagnoses formulated by the Pediatric Neuro-Psychiatrists with the scores on the CY-BOCS (using 16 as the cut-off score). The analysis revealed that it had a high Sensitivity (Se = 0.75) and Specificity (Sp = 1) for OCD.

The internal consistency indices uncovered a good internal consistency for both the Obsessions (α=0.81) and Compulsions scales (α=0.80).

Generally speaking, the correlations between the CY-BOCS sub-scales were very low (-0.25 < r < 0.22), except for the correlations between the Obsessions and Compulsions items connected to Time (r=0.41; p<0.01), those connected to Interference (r=0.41; p<0.01), and the Interference of the Obsessions item both with the Distress for Compulsions item (r=0.30; p<0.05) and the Compulsions Total score (r=0.33; p<0.05). The correlation between the total scores of the Obsessions and Compulsions scales was also very low (r=0.15; p>0.05) (**Table 4**).

**Table 4:** Intercorrelations of the CY BOCS sub-scales in the OCD sample studied (N=53).

CY-BOCS	Comp_1*Time occupied*	Comp_2*Interference*	Comp_3*Distress*	Comp_4*Resistance*	Comp_5*Control*	Comp*Total*
**Obs_1 *Time occupied***	0.41^**^	0.12	-0.002	-0.09	-0.12	0.13
**Obs_2 *Interference***	0.22	0.41^**^	0.30^*^	0.13	-0.01	0.33^*^
**Obs_3 *Distress***	0.13	0.15	0.15	-0.15	-0.16	0.08
**Obs_4 *Resistance***	0.12	-0.02	-0.22	0.03	-0.25	-0.04
**Obs_5 C*ontrol***	0.16	0.06	0.03	-0.13	-0.01	0.08
**Obs *Total***	0.27^*^	0.19	0.07	-0.05	-0.14	0.15

CY-BOCS, Children’s Yale-Brown Obsessive-Compulsive Scale; Obs, Obsessions; Comp, Compulsions.

Bold text refers to significative correlations at 0.05 or 0.001.

As far as the convergent validity between the CY-BOCS and the OCI-CV was concerned, our analysis showed that both the Obsessions and Compulsions scales of the CY-BOCS were positively correlated with the OCI-CV (r=0.55 and r=0.49, respectively; p<0.01). The Obsessions scale of the CY-BOCS was negatively correlated with the activities scale and the total competence score of the CBCL (respectively, r=-0.29 and r=0.34; p<0.05). Finally, only the Obsessions scale of the CY-BOCS was positively correlated with the total syndrome rating score of the CBCL (r=0.30; p<0.05) (**Table 5**).

**Table 5:** Correlations between the CY-BOCS and the OCI-CV and the CBCL in the OCD sample studied (N=53).

	OCI-CV*Total score*	CBCL*Activity*	CBCL*Social*	CBCL*School*	CBCL*School*	CBCL*CompetenceTotal score*
**CY-BOCS *Obs***	0.55^**^	-0.29^*^	-0.23	-0.27	-0.27	-0.34^*^
**CY-BOCS *Comp***	0.49^**^	-0.04	-0.25	-0.07	-0.07	-0.17

CY-BOCS Obs, Children’s Yale-Brown Obsessive-Compulsive Scale - Obsessions scale; CY-BOCS Comp, Children’s Yale-Brown Obsessive-Compulsive Scale - Compulsions scale; OCI-CV, Obsessive-Compulsive Inventory-Children Version; CBCL, Child Behavior Checklist.

Bold text refers to significative correlations at 0.05 or 0.001.

## Discussion

To our knowledge, this is the first study so far aiming to examine the CY-BOCS best model through the Bayesian approach considering an Italian sample.

Three two-factor models were examined. One was the Obsessions and Compulsions factor model (29) and the other two were the Disturbance and Severity factor models; in the first case, symptom frequency was loaded onto the Disturbance factor (29), and in the other, it was loaded onto the Severity factor (30). We also examined a highly debated single-factor solution that includes all obsessive and compulsive symptomatology (24–27). The Bayesian approach was used in view of its many advantages, including that of allowing the researcher to reach a better prospective likelihood of fit and to study and compare models even in small samples of patients. As far as our juvenile OCD group was concerned, our analyses showed that the two-factor model representing Obsessions and Compulsions was the best factorial structure of the CY-BOCS; the other three models examined showed inadequate fit.

These findings, which further confirmed the validity of the two-factor solution proposed by McKay et al. (29), have relevant implications for understanding the disorder, i.e., that Obsessions and Compulsions should be assessed separately. In fact, data analysis showed that a single-factor solution is unsuitable and that using it exclusively could lead to misinterpretation of the severity of the disorder. Not only that, but using it alone in a treatment context could lead to mistakes in interpreting intervention-related changes. Examining the two faces of the disorder separately would seem then to be a better approach to evaluating juvenile OCD.

These results are in line with one retrospective study (4) showing higher scores on the Compulsions scale of the Y-BOCS in those patients presenting an early-onset form of the disorder. They are also consistent with the findings of some studies showing that there tends to be a prevalence of Compulsions in these patients that often precedes the onset of Obsessions. In fact, some have reported that juvenile OCD cases are characterized by an onset of compulsive behavior alone (without obsessions) that may be indistinguishable from TIC (9, 10, 61–63).

As far as validity was concerned, the internal consistency of the CY-BOCS Obsessions and Compulsions scales was high, but in contrast to other studies in which the intercorrelations with the total score were high for both the Obsessions (0.77) and Compulsions (0.82) scales (64), in our sample, it was only moderate, especially for the Obsessions factor (r=0.47 for Obsessions; r=0.72 for Compulsions). Moreover, the intercorrelation between the Obsessive and Compulsive scales was very low (r ranging from -0.22 to 0.41), and only the time spent in compulsions was found to be correlated with the total score of the Obsessions scale (r=0.27). Likewise, the intercorrelation between the Obsessions and Compulsions scales was very low (r=0.15). However, the methodology used here did not enable us to make comparisons with other studies as far as construct validity was concerned: for example, Freeman etal. (64) found an internal consistency of 0.71 for Compulsions and only of 0.64 for Obsessions, while Storch etal. (30) found a moderate relation between Obsessions and Compulsions (r=0.49). Collectively, these findings show that the Obsessions and the Compulsions scales separately provide a clinically useful, reliable, and valid assessment of OCD severity in young children, suggesting that the two factors are distinct OCD constructs. This type of structure is useful to identify the frequent situations in children where obsessions are absent or there is no awareness of their presence. Assessing obsessions and compulsions together would result in an underestimation of OCD diagnosis, which could also lead to a lower probability of access to treatment for those children who would need it. Therefore, this factorial solution, instead, might include data regarding the presence of distinct factors at an early age and could allow early access to treatments, thus interfering with the characteristic tendency for it to become a chronic disorder (i.e., 12–15).

We cannot, however, entirely exclude the possibility that the immaturity of some of the children examined did not allow them access to the cognitive constructs linked to obsessions when, instead, the problem of compulsive behaviors was more evident to them and to others. Future studies with larger numbers of younger children over a wide age range will be able to clarify this point.

The study findings also showed that the Obsessions and Compulsions scales have a good convergent validity with the OCI-CV and confirm its usefulness.

Furthermore, the Obsessions and Compulsions scales were found to be negatively correlated with what parents said about their children’s competencies, as evaluated using the CBCL. In this case, the correlation with the Obsessions factor (r=-0.34) was stronger than that with the Compulsions one (r=-0.17). This was an unexpected result, as parents generally tend to be quite aware of the children’s difficulties. We could postulate that the parents’ evaluation could be mainly focalized on the cognitive resources indispensable for academic performance and that are affected by Obsessions.

As far as the sensitivity and specificity of the CY-BOCS were concerned, our findings show that they can be considered excellent. In the future, it would be interesting to investigate whether other (separate) cut-offs could be more useful in assessing the disorder’s severity and monitoring treatment.

Approximately 24.5% of the children in our sample attending the different mental health facilities participating in our study were not receiving any treatment a year after the diagnosis was formulated, and 26% were receiving only pharmacological treatment. Regardless of the reasons for this choice, treatment guidelines such as the National Institute for Clinical Excellence (65) recommend Cognitive Behavioral Therapy (CBT) for young people with OCD as the first line of treatment even if they have other comorbidities. Again, as far as the children studied here were concerned, 13.2% had left school or was struggling academically and requiring the assistance of a special needs teacher during classes. We are convinced that early assessment and appropriate intervention could lower that percentage (66) and, more importantly, could have a significant impact on the quality of life of the children and their families.

## Limits

These findings should be considered in light of the study’s limitations. First of all, as mentioned above and despite the presence of a parent during the interview, the youngest children participating in our study may not have been able to identify the cognitive aspects linked to obsessions. Moreover, our sample could be biased as we are not aware of how many individuals have refused to participate or have been excluded from the study based on exclusion criteria (e.g., due to having a neurological disorder).

In addition, as the results regarding the scales’ sensitivity and specificity are based on the data of a small subgroup of children in the TIC/TS group, the study findings should be interpreted cautiously, and further studies with larger samples are indeed warranted. Future studies could investigate temporal reliability, as it has not been assessed; these data would have important implications in the construct stability, considering this age range. Moreover, we would like to point out that more than half of the children in our sample had comorbidity with another diagnosis and, of 28 individuals (52.8%), 6 (11.3%) had a secondary diagnosis of TIC/Tourette Syndrome disorders. Therefore, it is not possible to exclude that the greater representativeness of the Compulsion aspect with respect to the Obsession one could be partially explained by tic-like behaviors, which often overlap with Compulsions. Finally, although the facilities participating in the study were distributed throughout the country, the sample cannot be considered representative of the Italian population since the sampling was on a voluntary basis. For all of these reasons, future studies are warranted.

## Conclusions

Our analyses showed that the best factorial structure of the CY-BOCS is a two-factor model representing Obsessions and Compulsions and suggest that the two scales of the CY-BOCS separately represent appropriate instruments for evaluating and monitoring the management of children with OCD. In any case, to better support the results of the present research, future studies should focus on a larger sample of children with OCD and without overlapping comorbidities.

## Data Availability Statement

The datasets generated for this study are available on request to the corresponding author.

## Ethics Statement

The studies involving human participants were reviewed and approved by approved by the Ethical Committees of the Department of General Psychology of the University of Padova and of all of the mental health facilities participating in the study. Written informed consent to participate in this study was provided by the participants’ legal guardian/next of kin.

## Author Contributions

CN conceived and planned the study. SP and FC contributed to sample preparation. MP contributed to the analysis of the data. All authors provided critical feedback and helped shape the research, analysis and manuscript.

## Funding

This work was carried out within the scope of the project “use-inspired basic research”, for which the Department of General Psychology of the University of Padova has been recognized as “Dipartimento di eccellenza” by the Ministry of University and Research.

## Conflict of Interest

The authors declare that the research was conducted in the absence of any commercial or financial relationships that could be construed as a potential conflict of interest.
